# Site-specific phosphorylation regulates the structure and function of an intrinsically disordered domain of the glucocorticoid receptor

**DOI:** 10.1038/s41598-017-15549-5

**Published:** 2017-11-13

**Authors:** Shagufta H. Khan, William A. McLaughlin, Raj Kumar

**Affiliations:** Department of Basic Sciences, Geisinger Commonwealth School of Medicine, Scranton, PA USA

## Abstract

Intrinsically disordered (ID) regions of the transcription factor proteins have much larger frequency of phosphorylation sites than ordered regions, suggesting an important role in their regulatory capacity. Consistent with this phenomenon, most of the functionally known phosphorylation sites in the steroid receptor family of transcription factors are located in the ID N-terminal domain that contains a powerful activation function (AF1) region. In this study, we determined the structural and functional consequences of functionally known phosphorylation residues (Ser203, 211, and 226) located in the human glucocorticoid receptor’s (GR’s) ID AF1 domain. We report the relative importance of each phosphorylation site in inducing a functionally active ordered conformation in GR’s ID AF1 domain. Our data demonstrate a mechanism through which ID domain of the steroid receptors and other similar transcription factors may adopt a functionally active conformation under physiological conditions.

## Introduction

Post-translational modifications such as phosphorylation may regulate protein functions of transcription factors by affecting their conformational dynamics leading to altered transcriptional activities^1,2^. Like many other transcription factors, the activities of nuclear hormone receptors including glucocorticoid receptor (GR) are regulated by multiple phosphorylation sites^3,4^. The GR is phosphorylated in the absence of glucocorticoid, with enhanced phosphorylation occurring when it is bound to agonist but not antagonist steroids^5^. In the human GR, functionally important phosphorylated residues (S203, S211 and S226) are located within AF1, and are conserved among species^5,6^. In spite of discovery that AF1 is quantitatively the major transactivation domain of the GR, we are only beginning to understand its structure-function relationship. Lack of this information has undermined our understanding of how GR transmits the transcriptional signal from ligand to specific gene(s). There are also reports suggesting that phosphorylation of Ser211 may affect GR conformation and thus alter receptor activity, and AF1 appears to be a main player in this process^7–9^. However, it is not yet known whether other phosphorylation sites (Ser203 and/or Ser226) also influence the structure and functions of the GR AF1. To understand how the GR transmits the transcriptional signal from ligand to specific gene(s), it is essential to gain the information regarding relative phosphorylation of each site on the structure and functions of the GR AF1.

The structure of AF1 of the steroid receptors has been difficult to determine because in solution it exists as an intrinsically disordered (ID) domain, frequently found in many transcription factors including steroid hormone receptors^10–13^. It has been reported that ID AF1 must undergo a disorder-order conformational transition for receptor’s optimal transcriptional activity^14–16^. Several studies have suggested that ID regions of the transcription factors have much higher frequency of known phosphorylation sites than ordered regions^1,17–19^. This is particularly true for the ID AF1 domain of steroid hormone receptors^16^. It has earlier been suggested that when phosphorylated at Ser211 site, GR AF1 may be structurally more stable and functionally more active^7,8,20^. However, the role of each phosphorylation site in regulating the conformation and functions of AF1 is not known. In this study, we report that GR’s ID AF1 domain undergoes conformational rearrangements leading to significantly enhanced AF1-mediated GR’s transcriptional activity in a site-dependent manner.

## Results

### Effects of site-specific phosphorylation on the conformation of ID AF1 Domain of the GR

**Figure 1:**
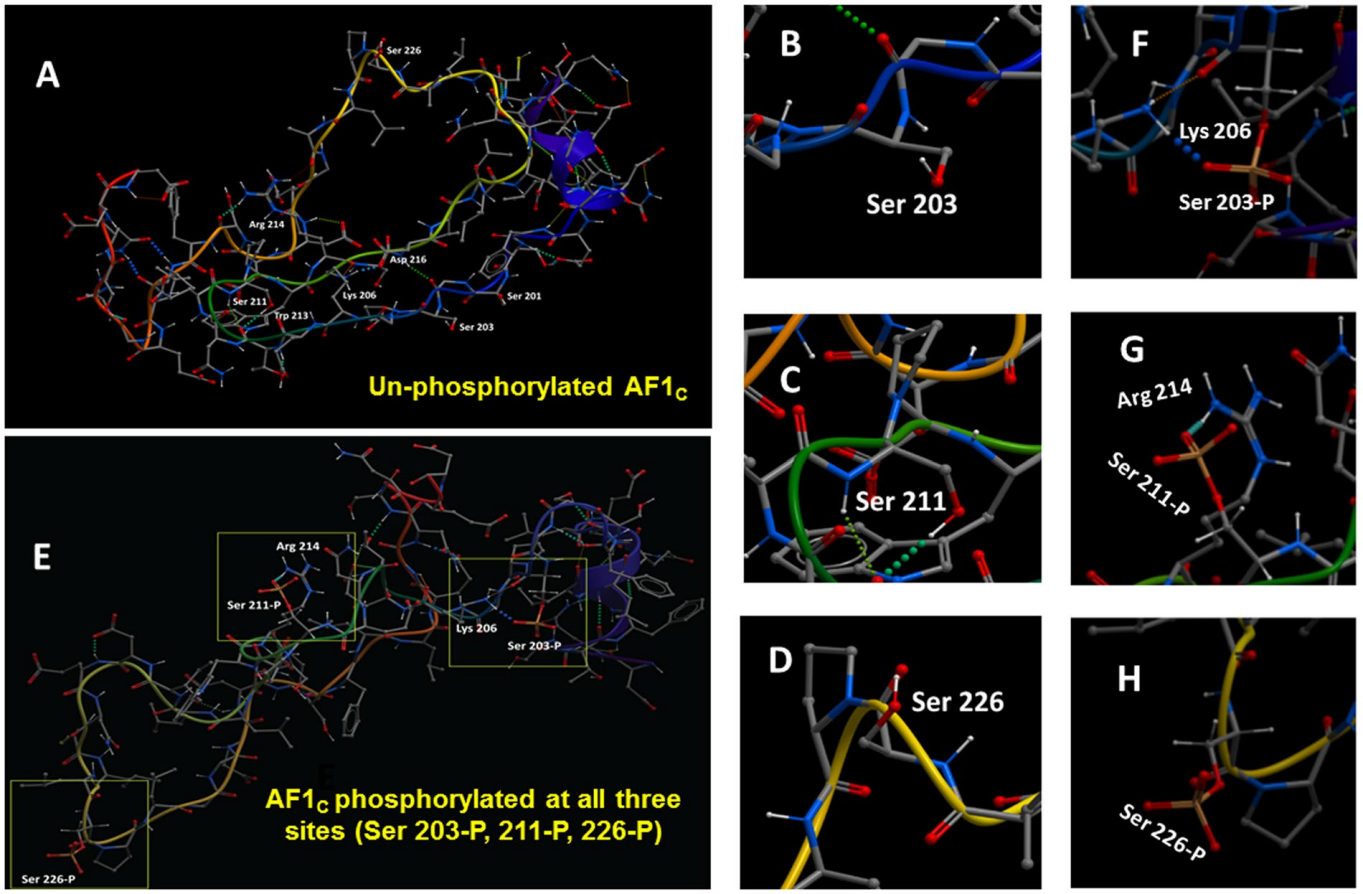
Simultaneous phosphorylation of all three sites (Ser203, 211 & 226) in the GR results in conformational changes in AF1. (**A**) Representative low-energy conformations of the non-phosphorylated AF1_C_ peptide. (**B–D**) close up view of non-phosphorylated Ser203, 211 & 226 residues, respectively. (**E**) Representative low-energy conformations of the AF1_C_ peptide when all three sites (Ser203, 211 & 226) are simultaneously phosphorylated. (**F–H**) close up view of phosphorylated Ser203, 211 & 226 residues and their respective binding to nearby residues. Phosphorylated Ser203 and 211 are shown forming a hydrogen bond network that is displayed as *dots* between donor and acceptor atoms.

To gain insight into the potential local structural changes in the GR AF1 upon phosphorylation at S203, S211, and S226 sites, we performed extensive global energy minimization simulations with AF1_C_ peptide (an AF1 core sub-domain), bearing phosphorylated vs. non-phosphorylated residues (Table S1). The predicted conformation of the non-phosphorylated AF1_C_ peptide exhibits a largely random and extended conformation (Fig. 1A–D), consistent with the expected biophysical measurements of the AF1 structure^8^. On the other hand, when all three residues (Ser203, Ser211, and Ser226) together are in the phosphorylated state, unique structural rearrangements are observed (Fig. 1E–H). In this case, phosphorylated Ser203 binds to Lys206 (Fig. 1E&F) whereas phosphorylated Ser211 binds to Arg214 (Fig. 1E&G), and phosphorylated Ser226 is solvent exposed (Fig. 1E&H). Such intramolecular interactions appear to induce local conformational rearrangements around these residues in the AF1_C_. The interactions are aided by hydrogen bonds, which are indicated by the spheres between the atoms in the models. As hydrogen bonds are one of the dominant drivers of macromolecular stability^21^, the observed charged hydrogen bond interactions, i.e. S203 to Lys206 and S211 to Arg214, may contribute to the formation of a more stable structure. We note here that the interaction of the phosphorylated Ser211 residue with Arg214 is comparable to that observed in the modeling study by Chen *et al*.^7^.

**Figure 2:**
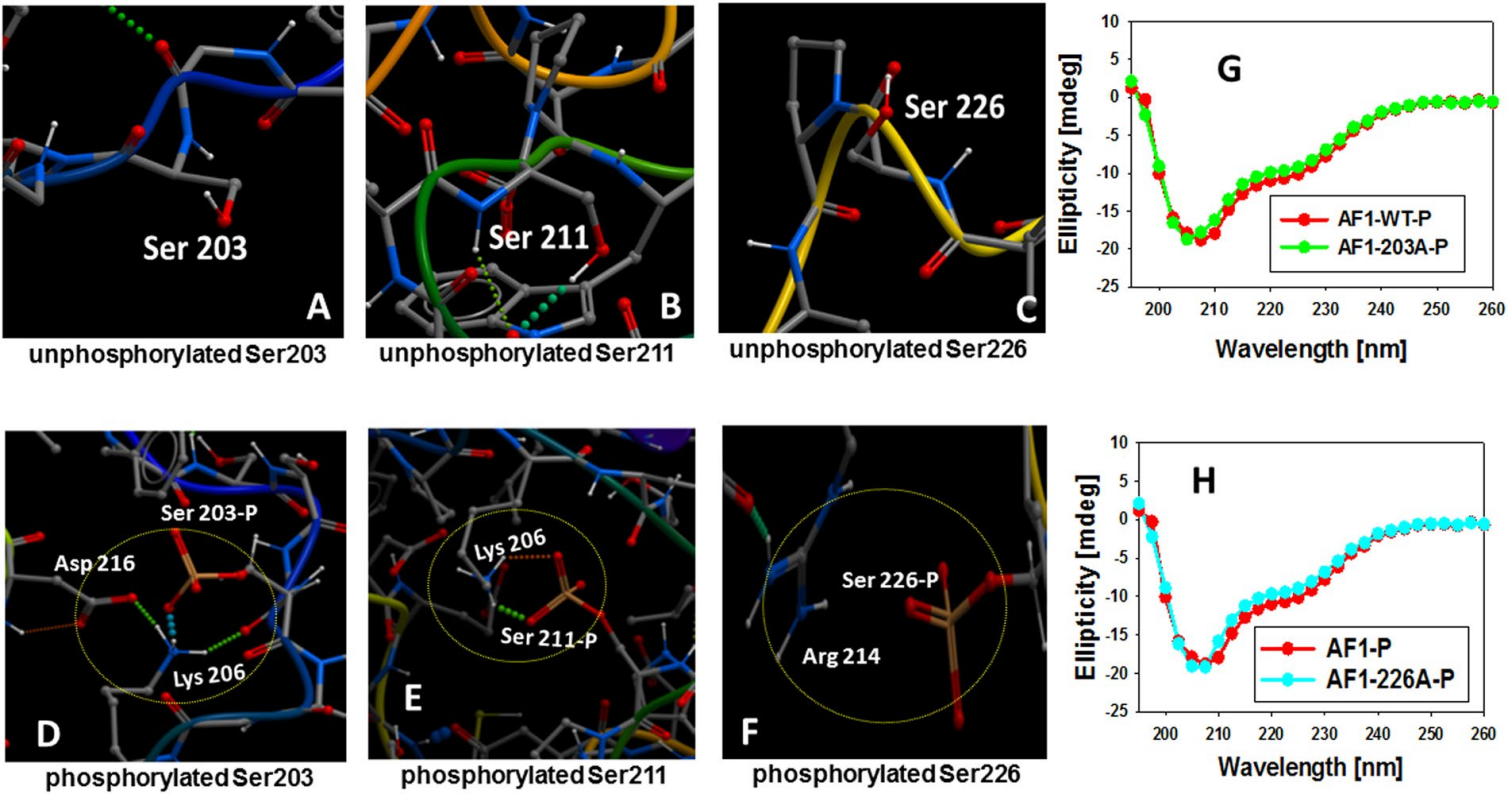
Effects of individual phosphorylation sites (Ser203, 211 & 226) on the structural changes in GR AF1. A close up view of non-phosphorylated Ser203, 211 & 226 residues (**A**–**C**), and individually phosphorylated Ser203, 211 or 226 residues (**D**–**F**), respectively. A comparison of far-UV CD spectra of phosphorylated AF1 peptide (WT) with Ser203A (**G**) or Ser226A (**H**).

We then determined the predicted conformation of the AF1_C_ peptide with each individual phosphorylated site (Figs 2 and S1–3). Shown in Figs 2A,D and S1 are the structural models of the non-phosphorylated vs phosphorylated AF1_C_ at Ser203. In this case, the phosphorylated Ser203 residue is still found to bind to positively charged Lys206, and there are additional supporting contacts of Lys206 with Asp216 and Lys206 with the backbone carbonyl of Ser203 (Figs 2A,D and S1). However, our experimental biophysical analysis suggests that this Ser203-Lys206 interaction does not result into any significant secondary structural rearrangements in AF1 (Fig. 2G), and we note that an interaction between Lys206 and Asp216 is observed in the model of the unphosphorylated polypeptide (Fig. 1A). Comparably, when Ser211 is phosphorylated, as shown in Figs 2B,E and S2, the negatively charged Ser211 residue now interacts with Lys206 to facilitate a conformational rearrangements to more ordered structure in AF1, consistent with our earlier studies showing that Ser211-phosphorylated AF1 adopts significantly higher secondary/tertiary structural elements in it^8^. In the case of phosphorylated Ser226, the residue appears to be primarily exposed to solvent (Figs 2C,F and S3). However, its proximity to Arg214 indicates that it participates in a charge-charge interaction to promote an inward bend in the structure, and this interaction seems to be weaker than those seen between the Ser203 or Ser211 and Lys206 under similar conditions, and does not appear to induce any significant secondary/tertiary structural rearrangements in AF1 (Fig. 2H). Further, biophysical analyses suggest that Ser211-Lys206 interaction induces a structural rearrangement in AF1 that affects the overall structure of the AF1_C_ (Figures S4,5). Together, these results suggest that individual phosphorylation of Ser203, Ser211 or Ser226 leads to an interaction of these negatively charged residues with positively charged residues (possibly due to addition of phosphate moiety). Interestingly, when phosphorylated, both Ser203 and Ser211 prefer to interact with Lys206 whereas Ser226 makes a weak contact with Arg214. Further, it appears that both phosphorylated Ser203 and Ser226 add to local structural re-arrangements whereas Ser211 phosphorylation leads to not only local structural re-arrangements but also to an overall more compact structure formation in the AF1_C_ peptide in general.

A further analyses of simultaneous phosphorylation of two sites together (Ser203/Ser211, Ser203/Ser226, or Ser211/Ser226) showed that when both Ser203 and Ser211 are phosphorylated, Ser203 becomes solvent exposed whereas Ser211 interacts with Trp213 (Figure S6). Similarly, when both Ser203 and Ser226 are phosphorylated, Ser203 interacts with Ser201 and Ser226 becomes solvent exposed (Figure S7). On the other hand when both Ser211 and Ser226 are phosphorylated, Ser211 interacts with Trp213 and not with Lys206, and Ser226 is solvent exposed (Figure S8). These observations provide evidence that different combinations of phosphorylation states appear to determine which residues bind intra-molecularly to the phosphorylated serine residues. We postulate the residue pair interactions of the phosphorylated serine residues can aid in determining which turns are adopted within the AF1_C_ and how the domain folds. The resulting different three-dimensional structures may promote different selective intermolecular bindings with other transcription factors to affect the expression rates of different sets of target genes.

**Figure 3:**
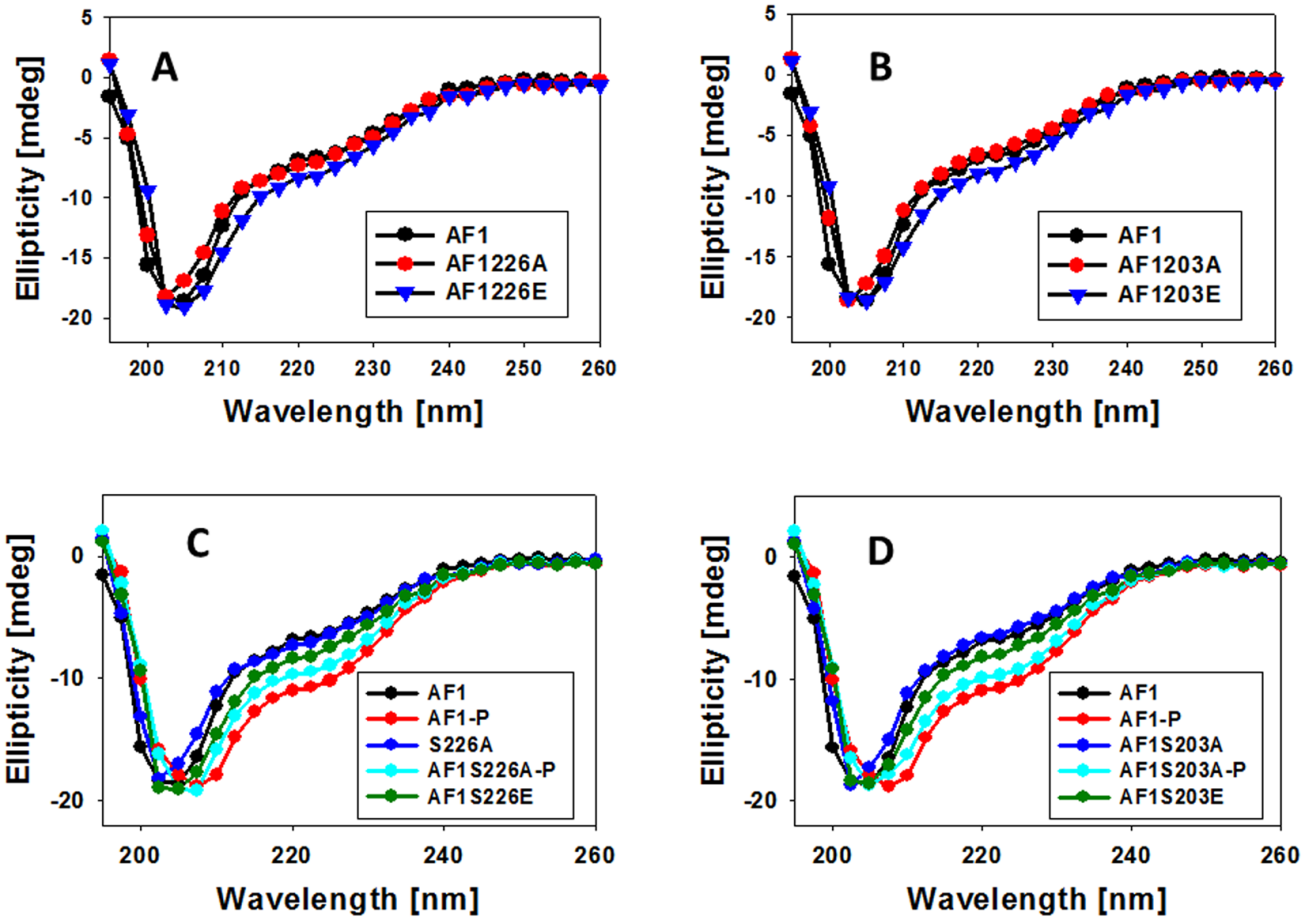
Far-UV CD spectra of the GR AF1 and mutants. Far-UV CD spectra of recombinant AF1, AF1-S226A and AF1-S226E (**A**); AF1, AF1-S203A and AF1-S203E (**B**). AF1 and AF1-S226A with or without p38 MAPK treated, and AF1-S226E (**C**); and AF1 and AF1-S203A with or without p38 MAPK treated, and AF1-S203E (**D**). AF1, non-phosphorylated AF1; AF1-P, phosphorylated AF1. Each spectrum represents an average of five spectra recorded, corrected for the contribution of the buffer and smoothed.

To test the effects of site-specific phosphorylation on the conformational changes in AF1, we recorded the far-UV CD spectra of non-phosphorylated and phosphorylated AF1. Under similar conditions, mutation of Ser203A or Ser226A do not show any significant secondary structural changes in non-phosphorylated AF1 (Fig. 3A,B) whereas when phosphorylated there is a significant increase in the secondary structure formation in AF1 (Fig. 3C,D), similar to previously reported which was lost with Ser211A mutant and was not completely mimicked by Ser211E mutant^8^. On the other hand AF1 mutants Ser203A or Ser226A showed significant structural changes, though slightly less than WT AF1 (Figs 3C,D and S9, S10). However, these increased secondary structural elements were largely mimicked by Ser203E or Ser226E mutants (Figs 3C,D & S9, S10) unlike that observed in case of Ser211E mutant^8^.

### Effect of site-specific phosphorylation on the AF1-mediated transcriptional activity of the GR

**Figure 4:**
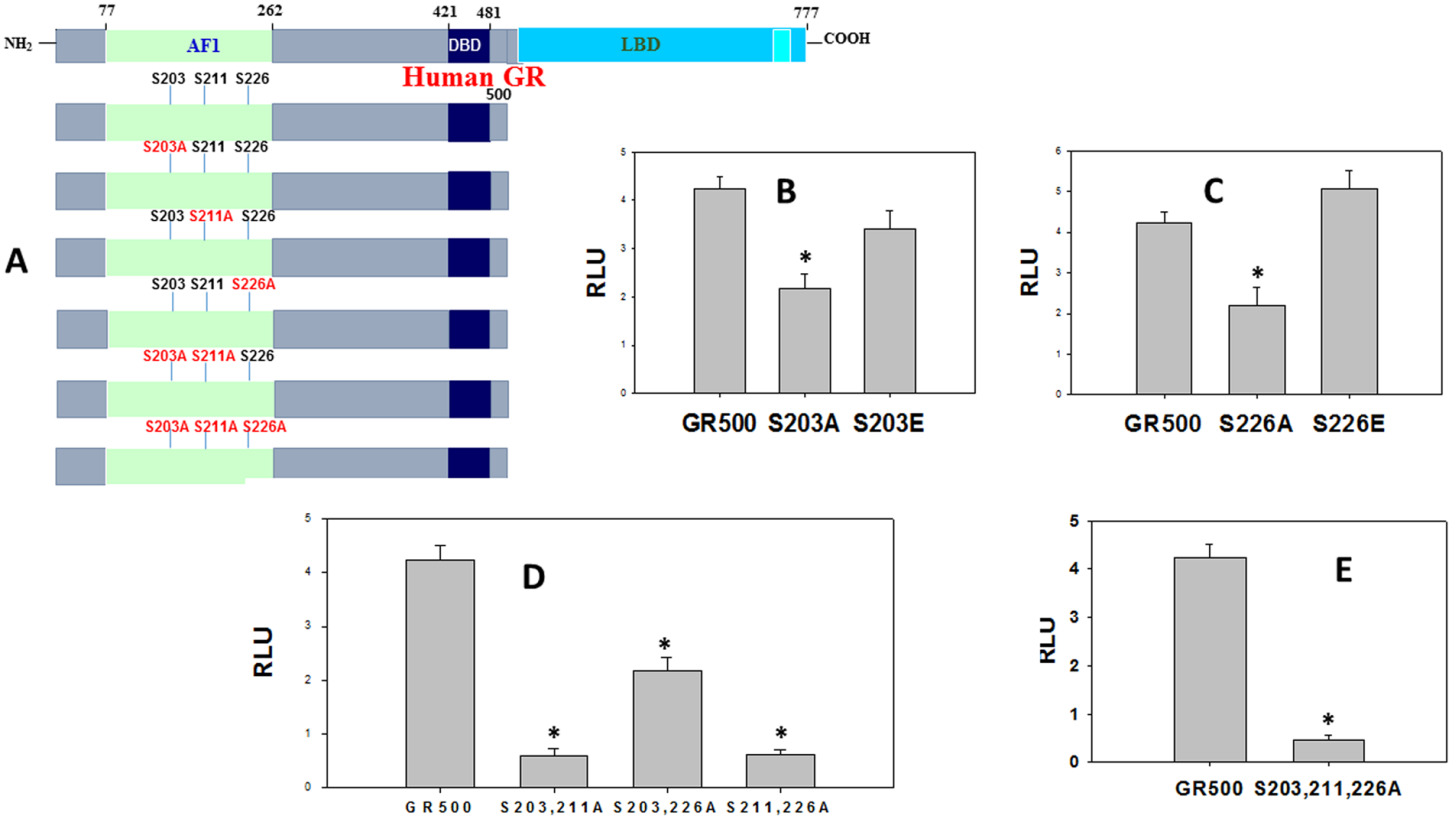
Phosphorylation-dependent GR AF1-mediated transcriptional activity of a promoter containing GRE. (**A**) Topological diagram showing various constructs of the human GR used for co-transfection studies to assess GRE-mediated AF1 activity by SEAP-based promoter-reporter assay in CV-1 cells. (**B–E**) Relative fold induction in cells treated with GR500, GR500-S203A, and GR500-S203E (**B**); with GR500, GR500-S226A, and GR500-S226E (**C**); with GR500, GR500-S203A/S211A, GR500-S203A/S226A, and GR500-S211A/S226A (**D**); and GR500 and GR500-S203A/S211A/S226A (**E**). The results are expressed as means ± the standard error. The levels of significance were evaluated by a two-tailed paired Student *t* test, and a *P* value of ≤ 0.05 was considered significant. Graphs were normalized to the transfection efficiency of each construct.

We tested the effects of site-specific phosphorylation-induced conformational changes on AF1-driven transcription using GR-responsive promoters in transient transfection-based secreted embryonic alkaline phosphatase (SEAP) reporter assay in GR-deficient CV-1 cells as described^8^. CV-1 cells were transfected with a GRE-SEAP promoter-reporter gene and a constant amount of GR500 expression with or without specific mutations (Fig. 4A & Table 2S). Lacking the ligand binding domain (LBD), GR500 is transcriptionally active without steroid and can significantly induce genes and/or apoptosis in cells similar to steroid-bound holo-GR^8,9^. As expected, GR500 alone significantly increased reporter activity compared to empty vector alone (Fig. 4). When GR500-S203A mutant was used, the reporter activity was significantly reduced (~50%), however this loss of activity was mostly recovered by GR500-S203E mutant (Fig. 4B). Experiments using GR500-S226A and GR500-S226E mutants showed similar results as those found in case of GR500-S203 mutants (Fig. 4C,D). It is important to note that under similar conditions, cells transfected with GR500-S211A mutant are reported to show a reduction of more than 75% of reporter activity, which is only moderately recovered by GR500-S211E mutant^8^.

To further understand the effect of phosphorylation on the GR-dependent transcriptional activity, we measured the effects of phosphorylation on the AF1-mediated transcriptional activity of the GR using various combinations of site-specific mutants. Cells transfected with double mutant GR500-S203A/S226A showed similar modest effects as in case of individual GR500-S203A or GR500-S226A (Fig. 4D). On the other hand, when GR500 contained S211A mutant in combination with either S203A or S226A, most of the activity was lost (Fig. 4D). In another set of experiments when all three phosphorylation sites were mutated together in GR500 (GR500-S203A/S211A/S226A) construct, there was no significant reporter activity observed (Fig. 4E). Together, these results strongly suggest that the effects of p38-mediated phosphorylation on the regulation of AF1-dependent GR action may be dependent upon the relative phosphorylation of individual Ser residues.

## Discussion

The mechanisms by which GR and other steroid receptors control gene expression pose a central problem in molecular biology and the role of their transcriptional activation domains in this complex process is of immense importance^16^. The GR protein is subject to multiple phosphorylation sites that regulate the function of the receptor^16^. These sites located in the ID NTD/AF1 domain contribute to multiple functions and the net effect of an individual phosphorylation depends on the activating kinase^7,8,22^. It has been suggested that ID AF1 domains of the GR and other steroid receptors with flexible structural dynamics play a critical role in dictating final outcome responsible for specific gene regulation. Post translational modifications such as phosphorylation elicit diverse effects on the biological functions of ID proteins by altering the energetics of their conformational landscape and by modulating interactions with other cellular components^18,19,23^ by stabilizing and/or inducing secondary structural elements^24–26^. Thus, signaling cascades that induce phosphorylation of the GR are important factors in determining the physiological actions of the GR. It has earlier been reported that p38-mediated phosphorylation of Ser211 plays an important role in regulating GR AF1’s conformation and subsequent glucocorticoid-induced apoptosis in leukemic cells^8,9^. However, the underlying mechanism that governs this important and yet complex phenomenon involving all major phosphorylation sites is not well understood. Our results provide a mechanism for how each phosphorylation site within its activation domain contributes to GR-mediated signaling through conformational rearrangements in otherwise ID AF1 domain.

Phosphorylation of Ser residue replaces a neutral hydroxyl (OH) group with a negatively phosphoryl group (PO_4_^2−^), which alters the steric, chemical, and electrostatic properties and thereby providing novel possibilities for intra- and inter- molecular interactions, including hydrogen-bonding networks. Under physiological conditions, the phosphoryl moiety is chemically different from the acidic amino acids (Asp and Glu) often used as phospho-mimics^26,27^. Although ID regions lack the ability to fold into stable 3-D structures on their own under physiological conditions, they undergo disorder-to-order conformational transitions under specific cellular environment^28^. Thus, changes in the surrounding physico-chemical environment can determine the ability of ID regions to fold into native-like species. In this context, site-specific phosphorylation-induced conformational changes due to altered physical/chemical properties of ID regions can be exploited for regulating biology. Although there are multiple phosphorylation sites critical for GR transcriptional activity (Ser203, Ser211, and Ser226), their respective role in driving GR functions is highly cell specific. Because p38 mitogen-activated protein kinase (MAPK) directly phosphorylates GR in different cell types in a stimulus- and cell-dependent manner, we used p38 MAPK for *in vitro* GR phosphorylation. Based on our results from the present studies combined with the results from earlier studies^8^, we propose that phosphorylation-induced conformational changes in the ID AF1 region of the GR are dependent upon the phosphorylation of individual site(s). It is interesting to note that phosphorylated Ser203 though appears to bind to nearby amino acids both when phosphorylation takes place alone or in combination with Ser211 or Ser226, yet fails to result into any significant structural changes unlike phosphorylation of Ser211 as reported earlier^8^. Further, functional data support that the moderate increases in AF1-mediated reporter activities of the GR due to site-specific phosphorylation are mostly reversed by Glu substitution in case of Ser203 and Ser226 but not with Ser211.

It is important to note that phosphorylation of Ser211 induces a more structured conformation in AF1_C_ with the peptides adopting higher helical contents (Figs  S4,5) similar to earlier findings for the full length AF1^8^. This suggests that phosphorylation of Ser211 confers structure to the GR AF1 domain including its AF1_C_ sub-domain. It is interesting that when all three sites are phosphorylated simultaneously, phosphorylated Ser211 exhibited a hydrogen bond interaction between the phosphate moiety on Ser211 and an adjacent residue, Arg214, which is part of the consensus sequence for CDK phosphorylation and is conserved among diverse species of the GR. This implies that flanking residues in the kinase consensus site might have evolved to contribute to the stabilization of local structure upon phosphorylation. Further, unlike, other two sites (Ser203 and Ser226), phosphorylated Ser211 makes contacts with Trp213 depending upon the status of phosphorylation of either Ser203 or Ser226 but not alone. We infer from the models that the interactions between Ser211 and Trp213 may result in hindering interactions between Ser211 and Lys206, as the Trp213 and Lys206 are predicted to be on the opposite sides of Ser211. Interestingly, mutation of Trp213 is known to significantly reduce receptor transcriptional activation^29^. We’ve also reported that the environment of the tryptophan (fluorescence steady state spectra) change upon Ser211 phosphorylation^8^. We therefore propose that the regions surrounding S211 are of critical importance to GR transcriptional activation perhaps because they contribute to the formation of the putative protein-protein interaction surfaces as reported earlier^8,30^. The structural and subsequent functional effects of phosphorylated Ser211 may thus be influenced by the relative phosphorylation of other two Ser residues. These findings support our previous studies where we found that p38-mediated phosphorylation of GR is involved in GR-mediated apoptotic events^31^ as well as p38 MAPK can *in vitro* phosphorylate all three sites^8^. These findings further support other reports, which have shown that the relative effects of Ser211 phosphorylation on GR-mediated target gene expression is dependent upon the phosphorylation status of other sites^7,32^. Mutational studies using various point mutations within the AF1_C_ have clearly established the role of important hydrophobic amino acids including Trp213 among others in the interaction between the AF1_C_ and coregulatory proteins and subsequent AF1-mediated GR’s transcriptional activity^33–35^.

Cell/tissue-specific effects of steroid receptors are tightly regulated through specific kinase(s)/phosphatase(s), and site-specific phosphorylation-induced conformational changes in AF1 and its subsequent effects on transactivation activities emerging from our studies combined with previously published studies^8^ may provide critical information on how different surfaces within the AF1 domain may be created and used to manipulate GR target gene expressions. Tissue-specific splice variants of the steroid receptors are located within the N-terminal domain, which can modulate AF1 conformation, giving rise to multivalent interactions and thereby regulating diverse cellular processes. Previous studies have illustrated the importance of synergistic effects of both AF1 and AF2 for target gene expression and our results from present studies provide a starting point for evaluating mechanisms for this selectivity, which are likely to involve relative effects of each phosphorylation site and of course other post-translational modifications^36^. Together, these results extend our previous studies^8^ by providing a detailed mechanism for cell/tissue specific effects of gene regulation by GR, and the role of each phosphorylation site in this process. We contend that understanding how each phosphorylation site of the receptor influenced by cell/tissue-specific kinases and coregulatory proteins combine to influence the structural dynamics of the receptor in general, and AF1 in particular will dramatically improve our ability to predictably perturb steroid receptor signaling and reduce undesired target activation. These strategies are general and can be employed within all ID segments of transcription factors and other signaling proteins in which ID sequences are found in abundance.

## Materials and Methods

### Protein expression and purification

Recombinant AF1 protein was expressed in *Escherichia coli* BL21(DE3) using recombinant vector pGEX-4T1-AF1 (Amersham Biosciences, Piscataway, NJ). AF1 mutants were generated by using a QuikChange site-directed mutagenesis kit (Stratagene, La Jolla, CA), and appropriate primers were designed. Recombinant proteins expressed in *E*. *coli* were induced with 1 mM IPTG (isopropyl-β-D-thiogalactopyranoside) for 4 h, lysed, and extracted. The bacterial extracts were loaded onto a glutathione-Sepharose column at 4 °C as described previously^12,13^. His-tagged AF1_C_ (residues 187–244) and mutants were expressed in *Escherichia coli* BL21(DE3), and purified on the Ni-NTA column (QIAGEN, Valencia, CA) using imidazole step-gradient as described^37,38^. AF1 and AF1_C_ proteins were further purified on a Resource Q column (Amersham Biosciences, Piscataway, NJ). Final protein purity of each protein was ≥ 95% as verified by presence of a single band on sodium dodecyl sulfate-polyacrylamide gel electrophoresis.

### Plasmids

The pGRE-SEAP vector (BD Biosciences, Palo Alto, CA) contains three copies of a GRE consensus sequence in tandem, fused to a TATA-like promoter (pTAL) upstream from the reporter gene for secreted alkaline phosphatase (SEAP). GR500 encodes amino acids 1 to 500 of the human GR, plus a five-residue nonspecific extension^39^. The GR500 variants were generated via PCR by using phCMV2-GR500 as the starting template and inserting the PCR fragments into pECFP-C1 (BD Biosciences) using XhoI/SmaI cloning sites. DNA sequencing was performed on all clones to confirm correct sequence.

### Peptide Simulations of AF1 core sub-domain

Models of the GR AF1_C_ were generated using the ICM-Pro software (MolSoft, La Jolla, CA). Eight different models were generated: 1) non-phosphorylated AF1_C_; 2) phosphorylated Ser203; 3) phosphorylated Ser211; 4) phosphorylated Ser226; 5) phosphorylated Ser203 & 211; 6) phosphorylated Ser203 & 226; 7) phosphorylated Ser211&226; and 8) phosphorylated Ser203, 211 & 226. The initial structure of the AF1_C_ was generated in the non-phosphorylated form using the I-TASSER server with default parameters^40^. Each model was subjected to 200 global optimization moves using biased probability Monte Carlo^41^ and 100 local minimization calls. That was followed by an additional 800 global optimization moves and 100 local minimization calls.

### *In vitro* phosphorylation assays

*In vitro* phosphorylation of recombinant AF1 or AF1_C_ was carried out by using a p38α/SAPKa assay kit (Upstate, Cell Signaling solutions, Lake Placid, NY). In each case, AF1 or AF1_C_ was incubated with active p38 MAPK for 2 h at 37 °C in a shaking water bath. The level of Ser203, Ser211 or Ser226 phosphorylation was determined by using immunoblot analysis with specific antibodies against phospho- Ser203, Ser211 or Ser226 and GR (Cell Signaling Technology, Beverly, MA) as described^8^.

### Circular dichroism (CD) spectroscopy

CD spectra of proteins (at 200 µg/ml with or without p38 MAPK in 5 mM Tris [pH 7.9] and 25 mM NaCl) were recorded on a Jasco-815 spectropolarimeter using a 0.1-cm quartz cell, with a bandwidth of 1.0 nm and a scan step of 0.5 nm, as described previously^12,13^. Each spectrum is representative of at least three independent experiments, corrected for the contribution of the buffer and smoothed.

### Cell culture and transient transfection

CV-1 cells (American Type Culture Collection) were grown at 37 °C in minimal essential medium with Earle’s salts (Invitrogen) supplemented with 10% (vol/vol) fetal bovine serum (Atlanta Biologicals, Norcross, GA). Cells were sub-cultured every 2 to 3 days. CV-1 cells were plated on a 24-well plate (1000 µl/well) 1 day before the transfection and transfected by using Lipofectamine 2000 (Invitrogen) according to the manufacturer’s protocol. Transfected cells were maintained at 37 °C in 5% CO_2_ and 95% air for the duration of the experiment (24 h).

### Reporter gene assays

CV-1 cells were cotransfected as described above with 0.15 µg of pGRE_SEAP reporter vector and 0.15 µg of pECFP-GR500 or mutant as described^8,32^. The total amount of DNA added was kept fixed at 0.3 µg by the addition of empty pECFP vector as and when needed. Medium (25 µl) was collected 24 h later and tested for the presence of SEAP (Great EscAPe SEAP detection kit; BD Biosciences) according to the manufacturer’s protocol. Experiments were performed at least three times, in triplicate. The data from different experiments were normalized to GR500 activity.

## Electronic supplementary material

Supplementary File
