# Supplementary material for: Site-specific phosphorylation regulates the structure and function of an intrinsically disordered domain of the glucocorticoid receptor
Source: Sci Rep. 2017 Nov 13;7:15440. doi: 10.1038/s41598-017-15549-5 (PMC5684351; doi:10.1038/s41598-017-15549-5)
Supplement: Supplementary file 1 — Supplementary File [file 41598_2017_15549_MOESM1_ESM.pdf]

Supplementary Information

Site-specific phosphorylation regulates the structure and function of an intrinsically disordered domain of the glucocorticoid receptor

Shagufta H Khan, William A McLaughlin, and Raj Kumar*

Department of Basic Sciences, Geisinger Commonwealth School of Medicine, Scranton, PA, USA

Table 1: A list of recombinant AF1 and AF1$_c$ proteins

Plasmid vector	Site of mutation	Type of mutation
AF1	none	Wild-type
AF1-S203A	203	Ser-Ala
AF1-S203E	203	Ser-Glu
AF1-S211A	211	Ser-Ala
AF1-S211E	211	Ser-Glu
AF1-S226A	226	Ser-Ala
AF1-S226E	226	Ser-Glu
AF1$_C$	none	Wild-type
AF1$_C$-S211A	211	Ser-Ala
AF1$_C$ -S211E	211	Ser-Glu

AF1$_C$ amino acids (187-244) sequences:

dqst fdilqdlefs sgspgketne spwrsdllid encllsplag eddsfllegn sned

AF1 amino acid (77-262) sequences:

gspgidlska vslsmglymg etetkvmgnd lgfpqqgqis lssgetdlkl leesianlnr stsvpenpks sastavsaap tekefpkths dvsseqqhlk gqtgtnggnv klyttdqstf dilqdlefss gspgketnes pwrsdllide ncllsplage ddsfllegns nedckplilp dtkpkikdng dqfivtd

Table 2: A list of mammalian expression vectors

Plasmid vector	Site of mutation	Type of mutation
pECFP-GR500	none	Wild-type
pECFP-GR500-S203A	203	Ser-Ala
pECFP-GR500-S203E	203	Ser-Glu
pECFP-GR500-S211A	211	Ser-Ala
pECFP-GR500-S211E	211	Ser-Glu
pECFP-GR500-S226A	226	Ser-Ala
pECFP-GR500-S226E	226	Ser-Glu
pECFP-GR500-S(203-211)A	203, 211	Ser-Ala, Ser-Ala
pECFP-GR500-S(203-226)A	203, 226	Ser-Ala, Ser-Ala
pECFP-GR500-S(211-226)A	211, 226	Ser-Ala, Ser-Ala
pECFP-GR500-S(203-211-226)A	203, 211, 226	Ser-Ala, Ser-Ala, Ser-Ala

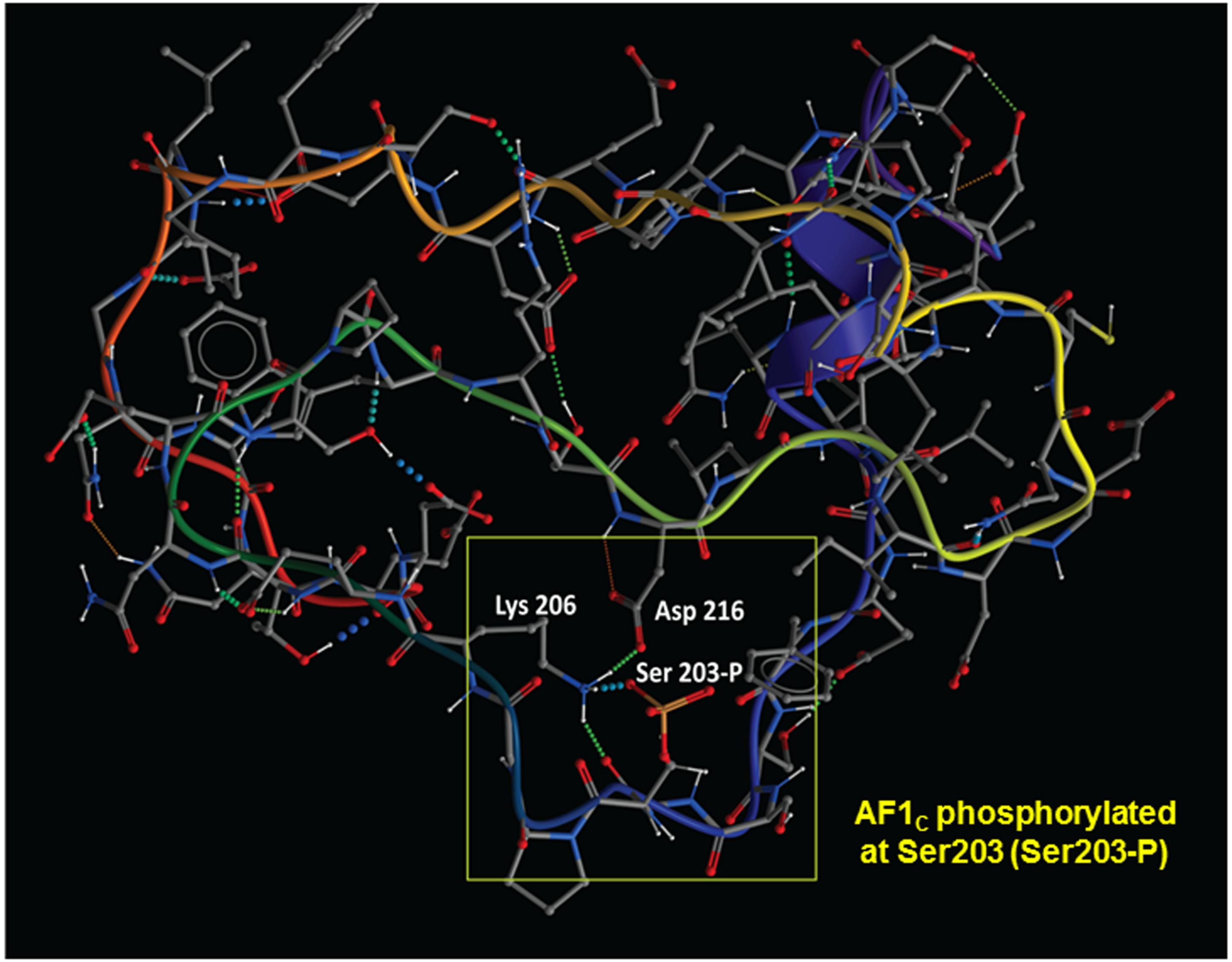

Supplemental Figure 1: A full view of AF1$_C$ (amino acids 187-242) of the human GR model with serine 203 phosphorylated. A model was initially built for the region in the unphosphorylated state using the I-TASSER software. Serine 203 was then phosphorylated using ICM-Pro. The resulting model was subjected to global optimization moves using biased probability Monte Carlo and local minimization calls as described in Materials and Methods. The resulting model shows an interaction between negatively charged phosphorylated Ser203 (Ser203-P) and Lys206 residue where a hydrogen bond is indicated with blue spheres (shown in the box).

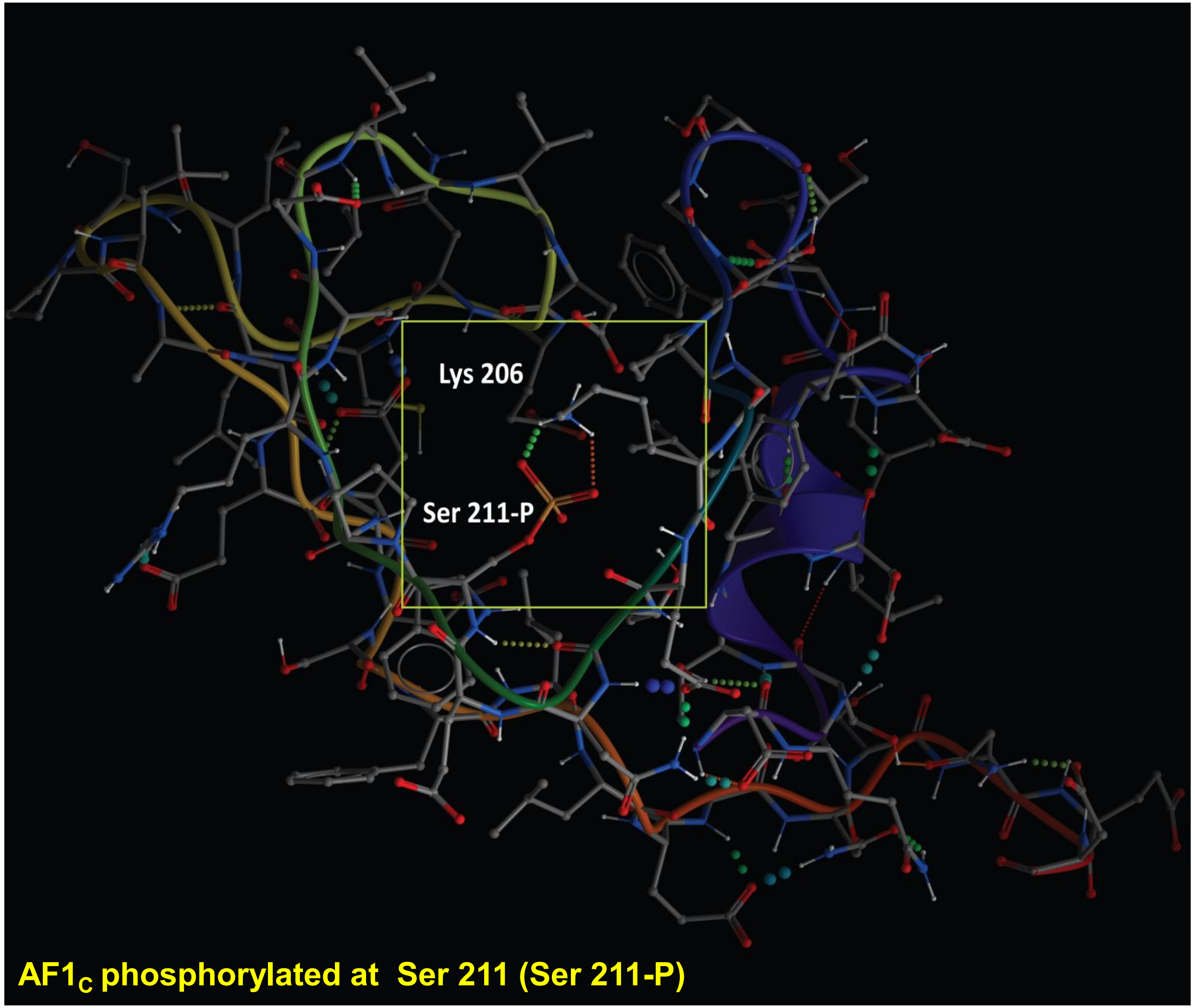

Supplemental Figure 2: A full view of $AF1_C$ (amino acids 187-242) of the human GR modeling the phosphorylation of serine 211. An interaction with two hydrogen bonds between negatively charged phosphorylated Ser211 (Ser211-P) and Lys206 residues is seen (shown in the box).

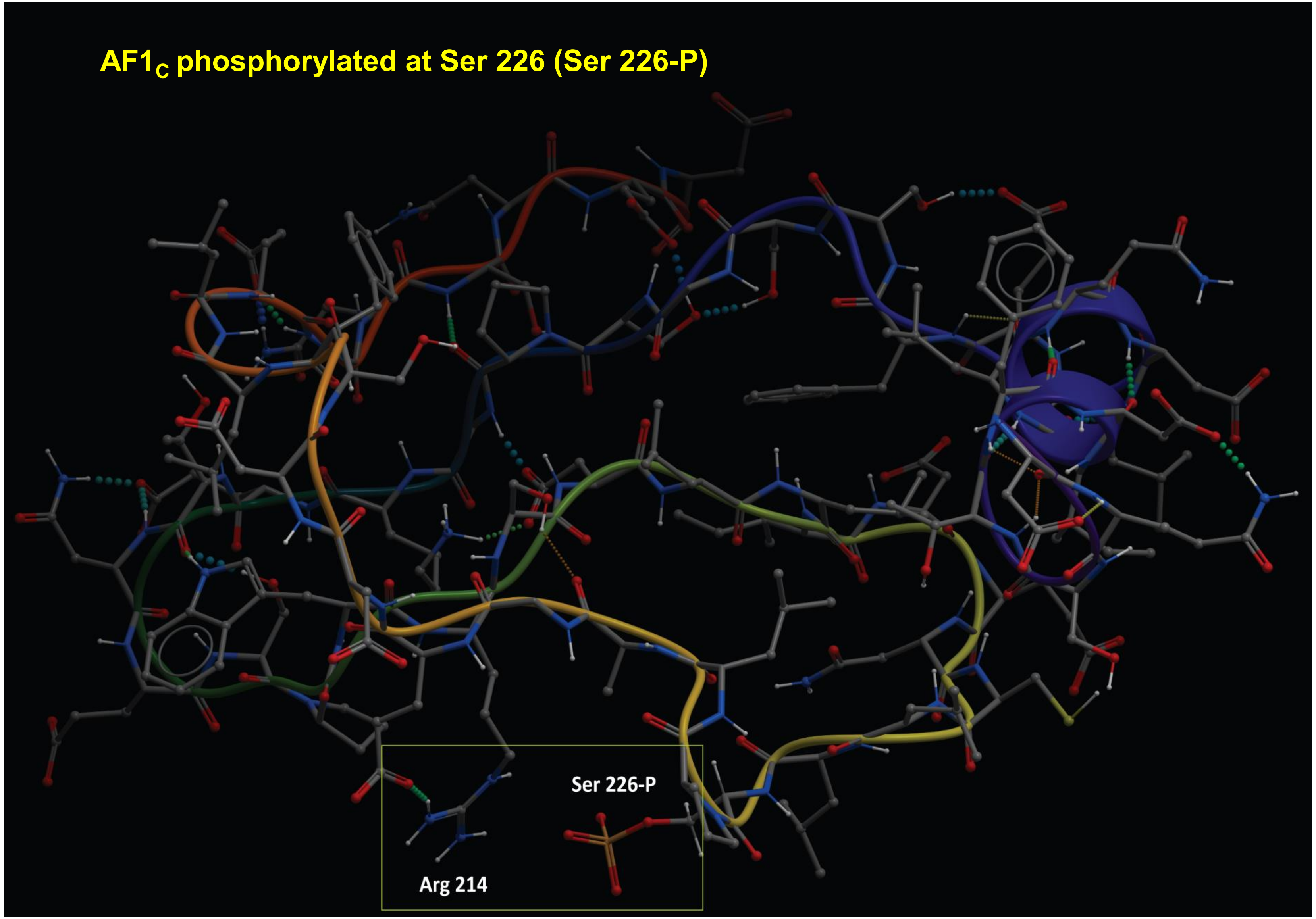

Supplemental Figure 3: A full view of AF1$_C$ (amino acids 187-242) of the human GR modeling the phosphorylation of Ser226. A close proximity, but no apparent hydrogen bonds, between the negatively charged phosphorylated Ser226 (Ser226-P) and Arg214 residues are seen (shown in the box).

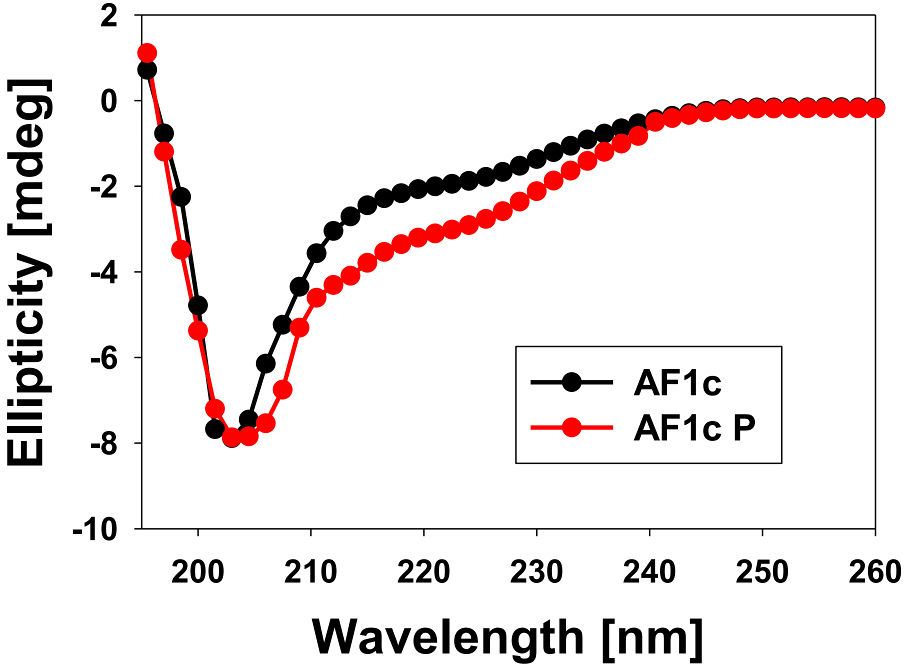

Supplemental Figure 4: Far-UV CD spectra of the recombinant GR $AF1_C$ with or without phosphorylated. $AF1_C$, unphosphorylated $AF1_C$; $AF1_CP$, phosphorylated AF1. Each spectrum represents an average of five spectra recorded, corrected for the contribution of the buffer and smoothed.

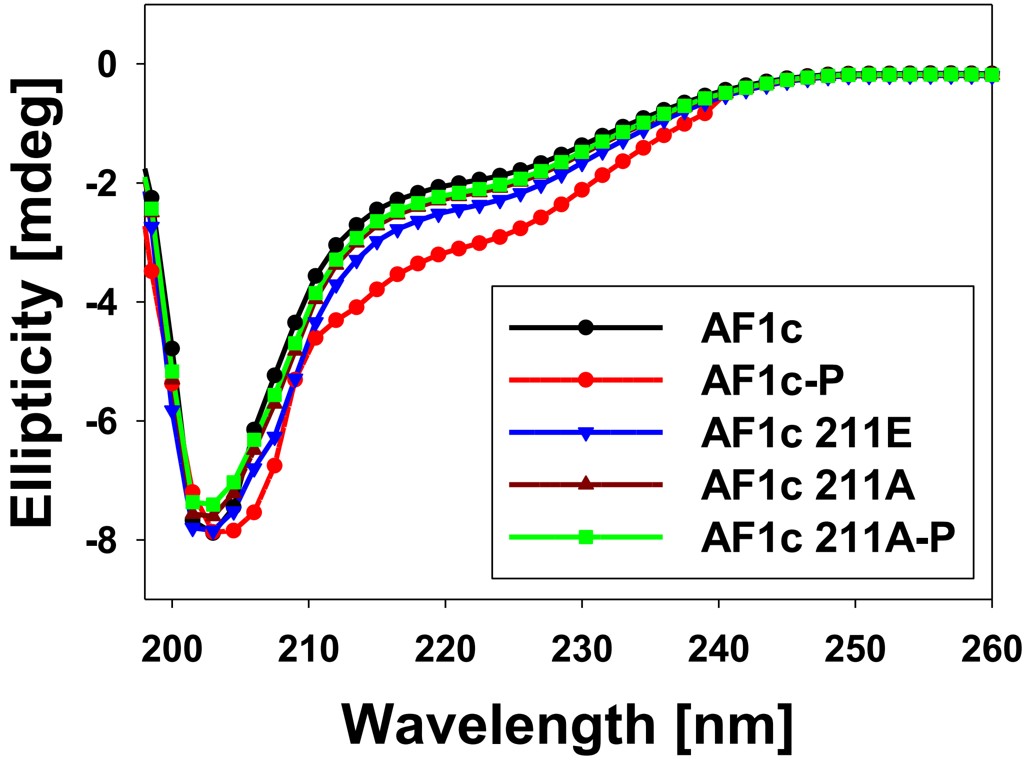

Supplemental Figure 5: Far-UV CD spectra of the GR $AF1_C$ (WT) and $AF1_C$-S211A with or without p38 MAPK treated, and $AF1_C$-S211E. $AF1_C$, unphosphorylated $AF1_C$; and $AF1_C$ P, phosphorylated $AF1_C$. Each spectrum represents an average of five spectra recorded, corrected for the contribution of the buffer and smoothed.

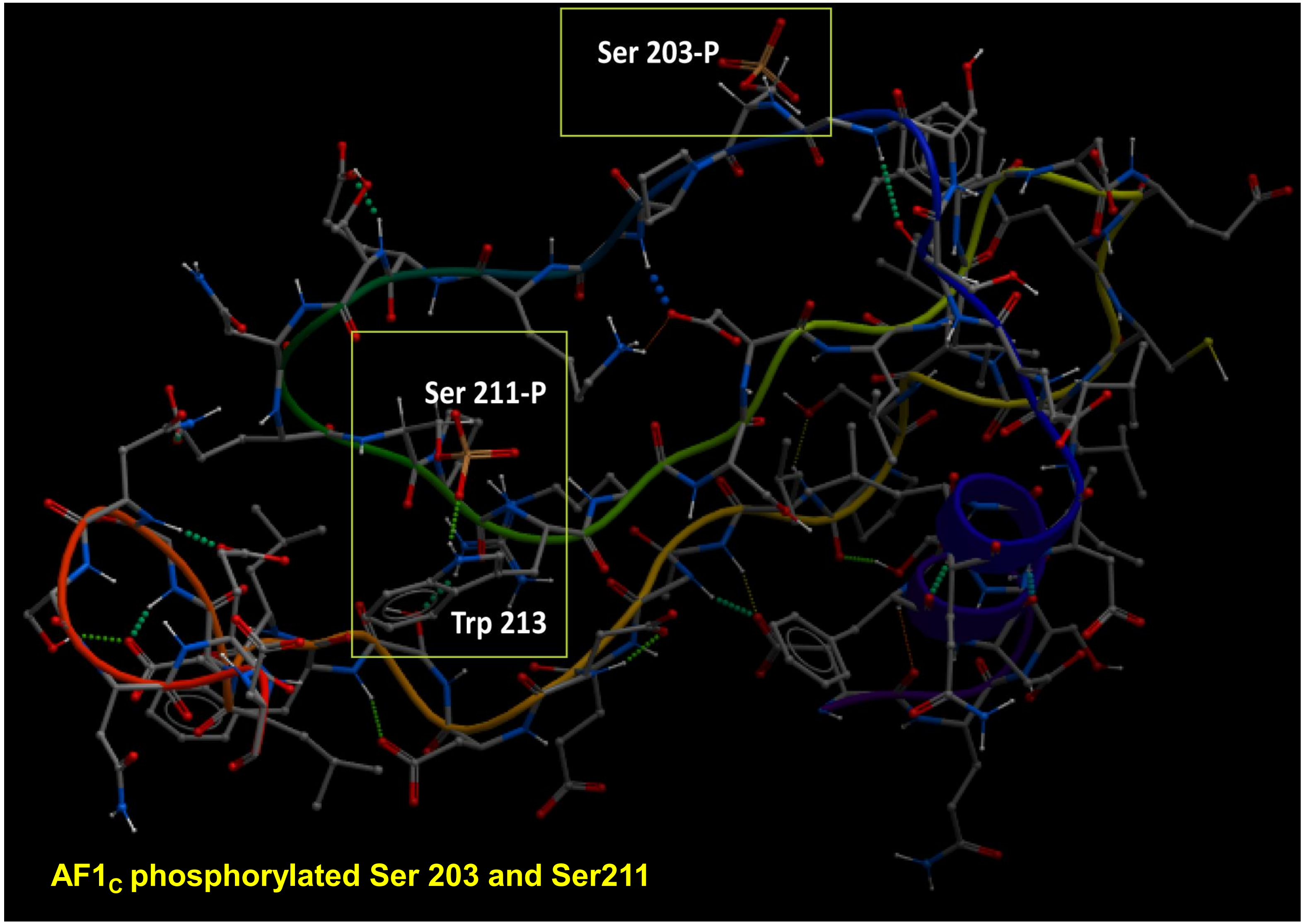

Supplemental Figure 6: A full view of AF1$_C$ (amino acids 187-242) of the human GR model with Ser203 and Ser211 residues simultaneously phosphorylated. In this case, phosphorylated Ser203 is seen to interact with solvent whereas Ser211 interacts with Trp213 (shown in the boxes).

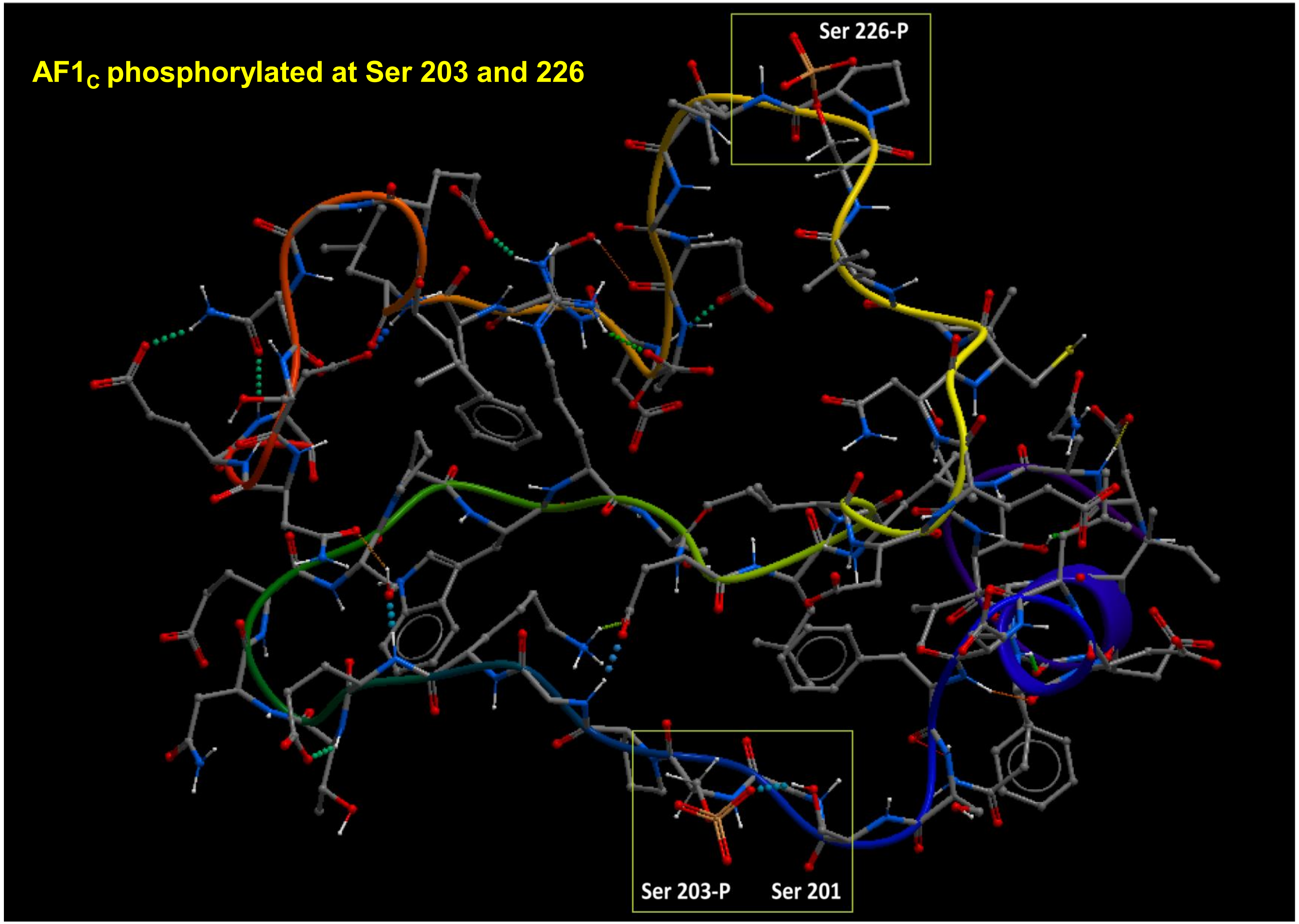

Supplemental Figure 7: A full view of $AF1_C$ (amino acids 187-242) of the human GR model with Ser203 and Ser226 residues simultaneous phosphorylated. In this case, phosphorylated Ser203 is seen to interact with Ser201 whereas Ser226 interacts with solvent (shown in the boxes).

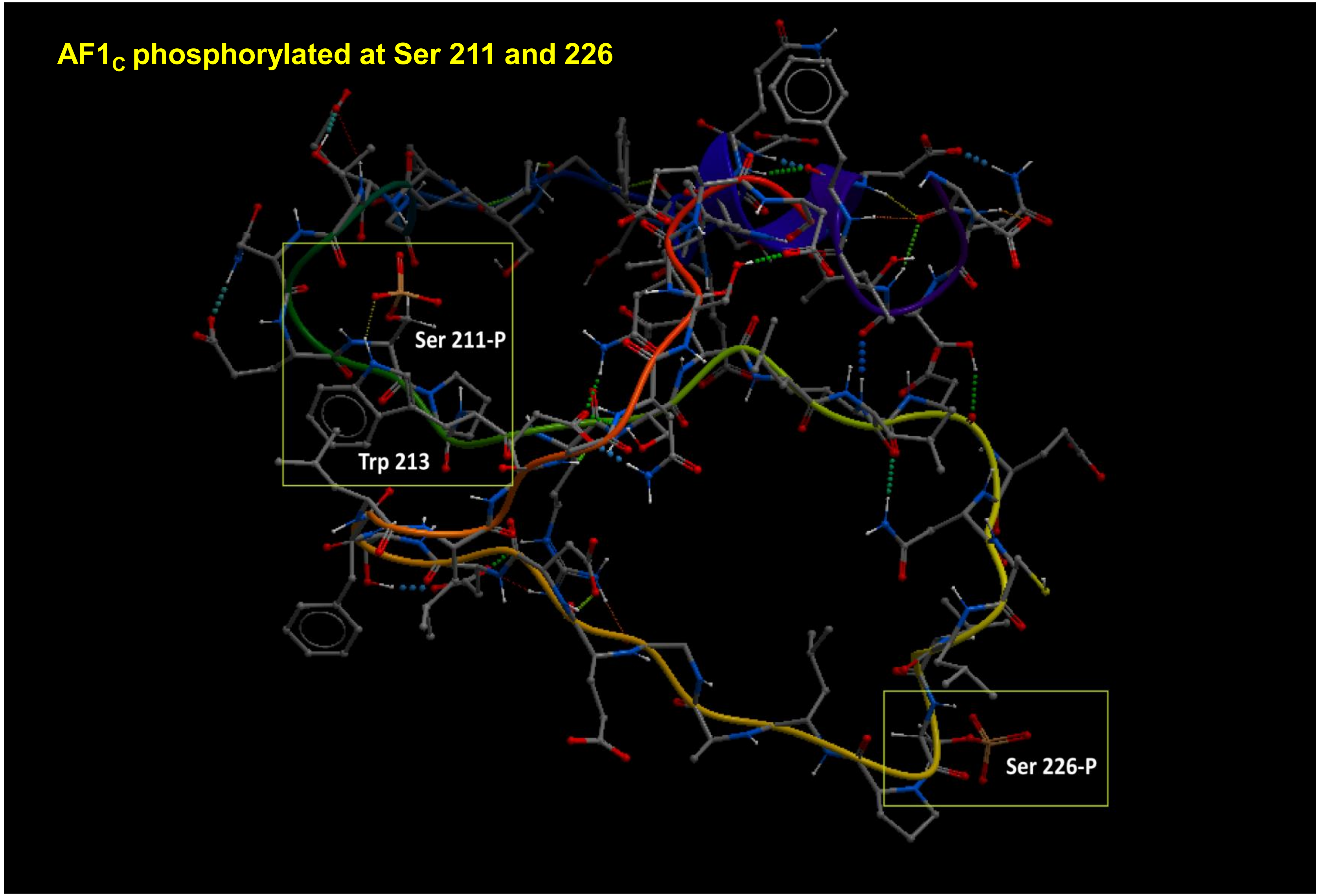

Supplemental Figure 8: A full view of AF1$_C$ (amino acids 187-242) of the human GR model with Ser211 and Ser226 residues simultaneously phosphorylated. In this case, phosphorylated Ser226 is seen to interact with solvent whereas Ser211 interacts with Trp213 (shown in the boxes).

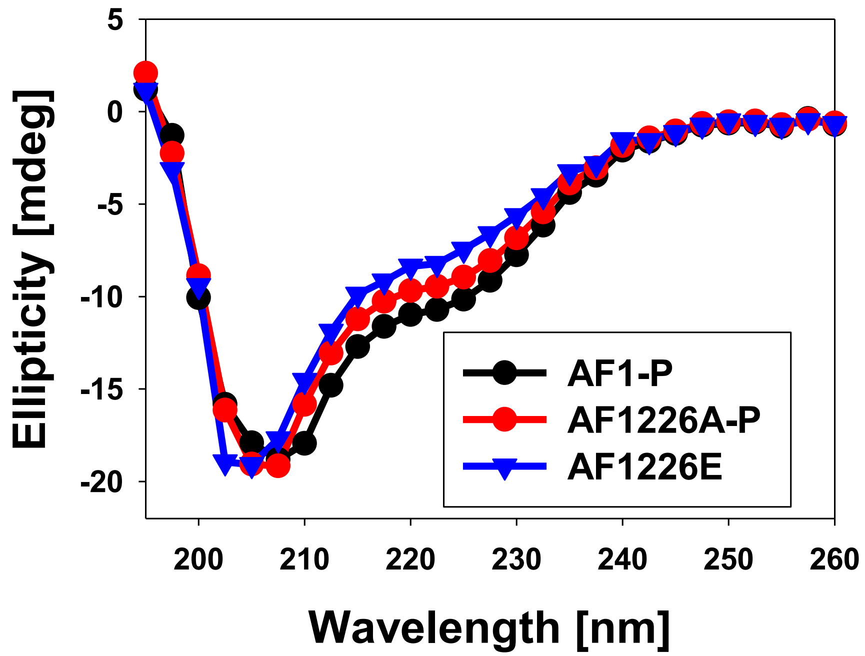

Supplemental Figure 9: Far-UV CD spectra of the GR AF1 (WT) and AF1-S226A treated with p38 MAPK, and AF1-S226E. AF1-P, phosphorylated AF1; AF1226A-P, AF1-S226A treated with p38 MAPK. Each spectrum represents an average of five spectra recorded, corrected for the contribution of the buffer and smoothed.

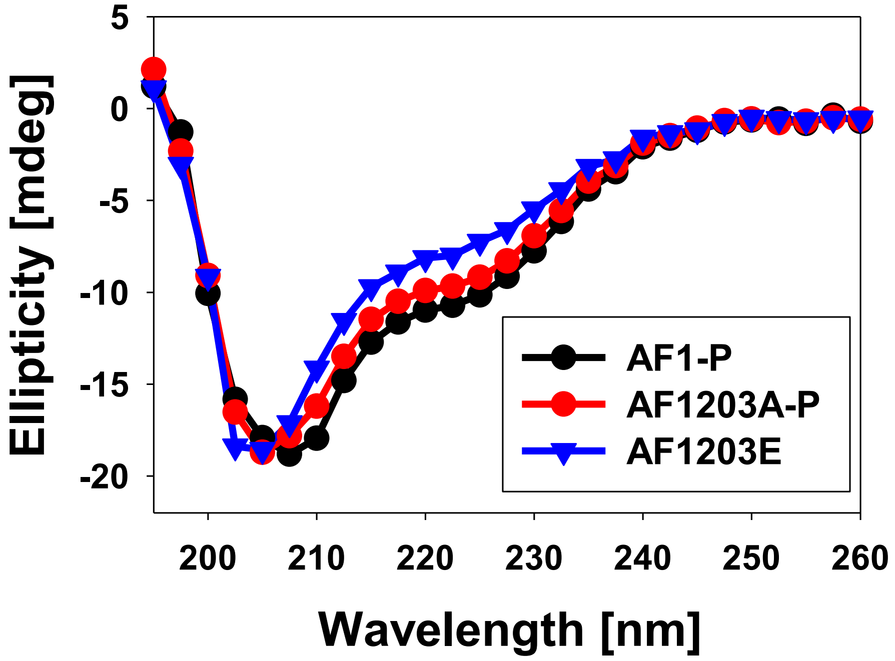

Supplemental Figure 10: Far-UV CD spectra of the GR AF1 (WT) and AF1-S203A treated with p38 MAPK, and AF1-S203E. AF1-P, phosphorylated AF1; AF1226A-P, AF1-S226A treated with p38 MAPK. Each spectrum represents an average of five spectra recorded, corrected for the contribution of the buffer and smoothed.
